# The Influence of Caregiver Distress and Child Anxiety in Predicting Child Somatization in Youth with Functional Abdominal Pain Disorders

**DOI:** 10.3390/children6120134

**Published:** 2019-12-03

**Authors:** Sarah C. Love, Constance A. Mara, Anne E. Kalomiris, Natoshia R. Cunningham

**Affiliations:** 1 Division of Behavioral Medicine and Clinical Psychology, Cincinnati Children’s Hospital Medical Center, Cincinnati, OH 45229, USA; love.63@wright.edu (S.C.L.); constance.mara@cchmc.org (C.A.M.); kalomiae@miamioh.edu (A.E.K.); 2 Department of Pediatrics, University of Cincinnati College of Medicine, Cincinnati, OH 45229, USA; 3 College of Human Medicine, Department of Family Medicine, Michigan State University Grand Rapids, MI 49503, USA

**Keywords:** pediatric, functional abdominal pain, anxiety, caregiver

## Abstract

Pediatric functional abdominal pain disorders (FAPD) are associated with adverse outcomes including increased somatization (e.g., heightened physiological sensations that include gastroenterological and non-gastroenterological symptoms) and increased functional disability. Caregiver distress and child anxiety are separately associated with the adverse outcomes of pediatric FAPD. However, the cumulative role of caregiver (i.e., stress, anxiety, and depression) and child psychological functioning (anxiety) in relation to adverse outcomes associated with FAPD, and particularly somatization, is unclear. Thus, the present investigation sought to examine the role of caregiver distress and child anxiety in relation to pain-related functioning (i.e., somatization, pain intensity, functional disability) in youth with FAPD. Data were gathered as part of a larger study examining a psychological treatment for youth with FAPD. Participants (ages 9–14) with FAPD completed measures of child anxiety, pain, and pain-related functioning. Caregivers completed a measure of caregiver distress (e.g., stress, anxiety, depressive symptoms). Pearson correlations revealed significant positive associations between child anxiety and child functional disability. Additionally, caregiver anxiety, child anxiety, and child somatization were all significantly and positively correlated with one another. Therefore, we assessed whether child anxiety may potentially mediate the relationship between caregiver anxiety and child somatization in this cross-sectional study. The indirect association between caregiver anxiety and child somatization via child anxiety was not significant. Future research including longitudinal designs to further understand the relationship between caregiver anxiety, child anxiety, and child pain-related functioning, would enhance understanding of how these potentially modifiable psychological factors may impact adverse outcomes of FAPD.

## 1. Introduction

Functional abdominal pain disorders (FAPD) are among the most common pain conditions in children and adolescents, with a median prevalence rate of 12% [1]. Pediatric FAPD are associated with adverse outcomes, including decreased quality of life [2] and increased impairment in social and academic functioning [3]. Additionally, FAPD is associated with high rates of functional disability (i.e., difficulty with daily tasks) and increased somatic symptoms and somatization [4,5,6,7,8]. Pediatric FAPD are also related to negative psychological outcomes in children, including higher rates of child anxiety and depression [9]. Furthermore, pediatric FAPD has also been found to be associated with poor parental mental health functioning, including increased rates of parental anxiety, depression, and stress [10,11].

Increased functional disability [12] and somatization [13] due to FAPD can be particularly debilitating for affected youth. Somatization is characterized as a heightened awareness of unpleasant physiological sensations, including gastroenterological (GI) and non-GI related symptoms. Indeed, youth with FAPD frequently report both GI and non-GI somatic symptoms, the latter of which includes headaches, dizziness, back pain, and sore muscles [8]. When these multiple somatic symptoms are not associated with an identifiable organic or medical cause, as is characteristic in youth with FAPD, the symptoms are considered somatization [5,6,7,8]. Somatization is an important predictor of outcomes for youth with FAPD as it has been found to be associated with increased pain severity over and above the presence of child anxiety [13], in addition to being associated with increased medical visits [14], and maintenance of pain into adulthood [6]. Further, FAPD is debilitating for a large portion of affected youth and is associated with increased rates of functional disability due to higher rates of school absences, increased sleep difficulties, and decreased engagement in extracurricular activities [12]. It is therefore critically important to study how potentially modifiable risk factors, such as child and caregiver psychological functioning, impact outcomes of FAPD (i.e., somatization, pain, and functional disability). This is critical given that these psychological and physical symptoms have the potential to persist into adulthood [15].

FAPD is associated with poorer child mental health functioning, including increased risk for anxiety and depressive symptoms. Anxiety disorders are highly prevalent in youth with FAPD, with 42% to 85% of youth with FAPD affected [16,17]. In terms of the relationships between child mental health functioning and adverse outcomes in youth with FAPD, the presence of anxiety symptoms in pediatric FAPD is associated with greater impairment and higher rates of adverse outcomes, including the maintenance of abdominal pain symptoms and increased functional disability that persists over time [18]. Additionally, previous research has demonstrated that baseline child anxiety and depressive symptoms are associated with specific trajectories of functional disability [18]. Specifically, individuals with FAPD with higher levels of anxiety, depression, lower perceived self-worth, higher impairment, and more negative life events represent a distinct long-term risk group, with higher long-term risk over a five-year period for both continued symptoms and impairment [18]. The presence of anxiety symptoms and other child psychological factors related to child anxiety, such as pain catastrophizing [19,20] and fear-avoidance [21], may also play a role, as both of these factors are related to increased functional disability. Although child anxiety is a significant contributor to pain-related impairment, it does not exclusively account for the poor outcomes associated with FAPD. Anxiety and FAPD are both associated with increased somatization and one study has found that somatization is a stronger predictor of pain symptoms than child anxiety in FAPD [13].

Although it is clear that child psychological functioning is related to outcomes in pediatric FAPD, considering additional factors such as the mental health functioning of caregivers may further elucidate risk factors for adverse child outcomes. Specifically, parenting behaviors and parental mental health functioning may influence the course of FAPD [11,20,21,22]. Parenting behaviors such as overprotection and minimization have been associated with increased disability in youth with FAPD, greater health care use, and higher medical costs for abdominal pain symptom management [20,22]. Additionally, increased child functional disability is significantly related to increased child and parent pain catastrophizing and higher levels of encouragement/monitoring and protection [19]. 

In terms of parental mental health factors (i.e., anxiety, depression, and stress), previous research has demonstrated that mothers and fathers of youth with FAPD are more likely than mothers or fathers of unaffected children to have anxiety symptoms [11,23,24]. Additionally, one study found that maternal and paternal anxiety in the first year of life predicted the development of pediatric FAPD at age six [11]. However, the relationship between parent anxiety and FAPD outcomes is unknown. Mothers of children with recurrent abdominal pain, but not fathers, have been found to report higher levels of depressive symptoms than mothers of healthy children [25]. Although parental or caregiver stress has not been studied extensively in youth with FAPD, previous research on chronic pain samples, including youth with recurrent abdominal pain, has found that parents of youth with chronic pain report higher levels of parenting stress [10]. Caregiver mental health may, therefore, impact youth with FAPD. Indeed, maternal mental health is related to poorer youth outcomes, with mothers with high levels of anxiety, depressive symptoms, and somatic complaints having children with poorer quality of life and greater use of ambulatory health services [11]. It would be valuable to understand how maternal anxiety impacts FAPD-related functioning (e.g., somatization) when also accounting for child-specific factors, such as child mental health functioning.

Research on the inter-relationships between parental distress, child anxiety, and child-pain related outcomes in youth with FAPD is limited. A previous study conducted by van Tilburg and colleagues in 2015 examining familial aggregation of irritable bowel syndrome (IBS) in parents diagnosed with IBS and their children, and relationships between maternal and child somatization, psychological distress, anxiety, and depression, found that maternal distress (e.g., anxiety, depression, and somatization) and child’s distress were related. While a comprehensive study, it is noteworthy that this research examined children of caregivers diagnosed with IBS, not children with FAPD diagnoses per se, and did not examine additional pain-related outcomes such as functional disability [26]. A prior study from our research laboratory found that child anxiety thoughts specific to pain (i.e., pain catastrophizing) mediated the association between parent responses to their child’s pain and child functional disability in youth presenting to a pediatric pain clinic with FAPD conditions [19]. However, the influence of these factors on child somatization was not explored, nor was the role of child anxiety more broadly examined as a predictor of pain and pain-related disability in youth [27,28,29]. It is important to study the role of child anxiety in culmination with caregiver anxiety because our research group has consistently found that general child anxiety is associated with increased pain and disability and poorer treatment response [30]. Therefore, there is a critical need for research to examine how caregiver distress and child anxiety simultaneously impact child outcomes related to FAPD including somatization, functional disability, and pain intensity. 

At present, it is unknown how caregiver distress and child anxiety cumulatively influence pain-related outcomes such as pain, functional disability, and somatization in youth with FAPD. Thus, the present investigation sought to examine the role of caregiver distress (e.g., stress, anxiety, depression) and child anxiety in relation to FAPD-related outcomes (e.g., somatization, pain levels, functional disability) in youth with FAPD. The current study aimed to (1) examine relationships between caregiver distress, child anxiety, and child FAPD outcomes, and (2) examine the role of child anxiety as a potential mediator in the association between caregiver distress and child FAPD outcomes. It was hypothesized that higher levels of caregiver distress, child anxiety, and poorer FAPD outcomes (e.g., somatization, pain levels, and disability) would be related, and that greater levels of child anxiety would indirectly link higher caregiver distress to poorer child pain-related outcomes in youth with FAPD, and thus serve as a potential mediator. 

## 2. Materials and Methods

### 2.1. Participants

Participants included youth with FAPD between the ages of 9–14 presenting for treatment at several pediatric gastroenterology clinics at Cincinnati Children’s Hospital Medical Center in Cincinnati, OH. Youth were recruited as part of a larger research study examining the effect of stepped care cognitive behavioral therapy for pediatric FAPD. The child’s primary caregiver was also recruited to participate in the study. During the screening process for study eligibility, the physician completed a checklist based on Rome IV criteria to confirm a diagnosis of FAPD [31].

### 2.2. Procedures

Data were collected (between 2015 and 2017) by a trained clinical research coordinator during a pediatric gastroenterology office visit at Cincinnati Children’s Hospital Medical Center. All study procedures were approved by the hospital’s Institutional Review Board. After receipt of informed consent and assent from both the child and the primary caregiver, youth completed self-report measures of anxiety, pain, and pain-related disability. Caregivers completed background and demographic information, in addition to a measure of their own psychological distress (See Questionnaires Section 2.2.1 for additional details).

#### 2.2.1. Questionnaires 

##### Background and Demographics

Participants’ primary caregivers were asked to complete demographic information such as child age, race, and ethnicity. See Table 1.

##### Caregiver Distress Measure

Depression Anxiety Stress Scales (DASS-21, Parent Report) assesses symptoms of depression, anxiety, and stress in adults (caregivers) using a 21-item questionnaire [32]. Each item is rated on a 4-point Likert scale ranging from 0 (did not apply to me) to 3 (applied to me very much or most of the time). The DASS-21 is comprised of several scales with seven items each. The Depression scale assesses dysphoria, hopelessness, lack of interest/involvement, anhedonia, and inertia. The Anxiety scale assesses autonomic arousal, situational anxiety, and subjective experience of anxious affect. The Stress scale captures levels of chronic non-specific arousal, including difficulty relaxing, nervous arousal, and being easily upset/agitated, irritable/over-reactive and impatient. The internal consistency of the DASS-21 for the current sample was excellent at 0.93.

##### Child FAPD and Associated Symptoms Measures

The Screen for Child Anxiety-Related Disorders (SCARED, Child Report) is a widely used instrument for examining anxiety symptoms in youth [33]. It has 41 items, and has been validated for use in children ages 8–18. The SCARED has been validated in pediatric pain samples including youth with functional abdominal pain conditions [34,35]. Youth are asked to report frequency of anxiety symptoms over the past three months. Responses include: “not true”, “sometimes true”, and “often true”. Total scores range from 0 to 82, with higher scores reflecting greater levels of anxiety. A score of 25 or greater is considered clinically elevated anxiety, and elevated SCARED scores, in general, are associated with increased pain and disability in youth with FAPD [28,29]. The internal consistency of the SCARED for the current sample was 0.94, which is considered excellent. 

The Children’s Somatization Inventory (CSI-24, Child Report) is a 24 item questionnaire that assesses child somatization, which is the perceived severity of 24 nonspecific somatic symptoms. The items included in the CSI are from the symptom criteria for somatization disorders defined by the DSM-III-R. The response format is a 5-point scale ranging from 0 (not at all) to 4 (a whole lot). Examples of CSI items include GI pain in addition to non-GI symptoms such as headaches, low energy, dizziness, and chest pain. Total CSI scores, obtained by summing all item ratings, can range from 0 to 140. The internal consistency of the CSI for the current sample was 0.91, which is considered excellent.

The Functional Disability Index (FDI, Child Report) is a self-report questionnaire is used to assess difficulty in completing various activities due to health symptoms in children ages 8 and older with chronic health conditions [36]. Available responses for each of the 15 items range from 0 (no trouble) to 4 (impossible). Item responses are summed to create a total disability score (range = 0–60) which is interpreted as follows: no/minimal disability = 0–12, moderate disability = 13–29, severe disability = 30+ [37]. The internal consistency of the FDI for the current sample was 0.83, which is considered very good.

The Visual Analog Scale (VAS, Child Report) for pain intensity is a 10 cm horizontal line anchored with the words “no pain” and “worst pain” to measure average pain intensity over the last two weeks [38].

#### 2.2.2. Data Analytic Plan 

##### Missing Data Handling 

A very small amount of missing data was present, with complete data available for over 94% of the sample. The remaining portion of the sample were missing single values on either the SCARED (*n* = 5) or the CSI (*n* = 1). Missing data were handled using maximum likelihood estimation.

##### Data Analytic Plan 

First, bivariate correlations were conducted in SPSS version 25 to understand the associations between caregiver distress (DASS Anxiety, DASS Depression, DASS Stress), child anxiety (SCARED), and child-associated outcomes (VAS, FDI, and CSI) of FAPD. 

Second, indirect effects models were examined for variables demonstrating significant correlations between caregiver distress, child anxiety, and child-associated outcomes. That is, based on significant correlations between caregiver distress, child anxiety, and child FAPD outcomes, we explored whether child anxiety mediated the association between caregiver distress and child FAPD outcomes. This model was based on our prior work, which found that child pain catastrophizing (a cognitive manifestation of child anxiety) mediated the association between parent responses to pain and child FAPD outcomes [19]. For the current study, we specifically investigated if there was an indirect relation between caregiver distress (DASS Anxiety, Depression, Stress) and child FAPD outcomes (CSI, FDI, and VAS) through child anxiety (SCARED). If age and gender were significantly related to any variables in the proposed model, they would be controlled for in subsequent analyses. 

For significant correlations, analyses were conducted using Mplus version 8.2 to evaluate each of the individual paths of the proposed indirect effects model with maximum likelihood estimation. Child FAPD outcome (i.e., somatization, functional disability, or pain) was regressed on caregiver distress as the predictor (i.e., anxiety, depression, stress), child anxiety (mediator) was regressed on caregiver distress (predictor), and child outcome was regressed on child anxiety while controlling for caregiver distress. The indirect effect was also tested using the model indirect function in Mplus with 1000 bootstraps. 

## 3. Results

### 3.1. Participant Characteristics

Participants included 90 youth (38 males, 52 females) between the ages of 9 and 14 (mean age = 11.6). The current sample population was predominantly Caucasian (see Table 1 for additional demographic characteristics). Participants also included 90 caregivers (10 males, 80 females). The caregiver sample was comprised of 81.1% mothers, 7.8% fathers, 7.8% grandmothers, 2.2% foster parents, and 1.1% grandfathers. All participating caregivers were legal guardians who also identified as the primary caregiver of the participating child at the time of the study.

### 3.2. Primary Analyses

Child age was significantly and positively correlated with child FDI and DASS Depression scores. Gender was not significantly associated with any of the variables examined. 

First, bivariate relations were examined to determine which indirect effects models to investigate (See Table 2). As expected, caregiver distress measures were significantly correlated with each other (all *r*s > 0.61). The child FAPD outcome variables of FDI and VAS were positively associated with one another, but were not significantly correlated with scores obtained on the DASS Anxiety, Depression, or Stress Scales. DASS Anxiety scores and CSI scores were significantly correlated with one another, but there was no significant association between DASS Depression or Stress and CSI. Further, DASS Anxiety scores were significantly correlated with scores on the SCARED. Scores on the SCARED were positively associated with child CSI and FDI scores. Due to these correlations, only CSI was examined as an outcome variable in the indirect effects model. Since none of the variables in the final model were related to age or gender, these variables were not controlled for in subsequent models. 

**Figure 1:**
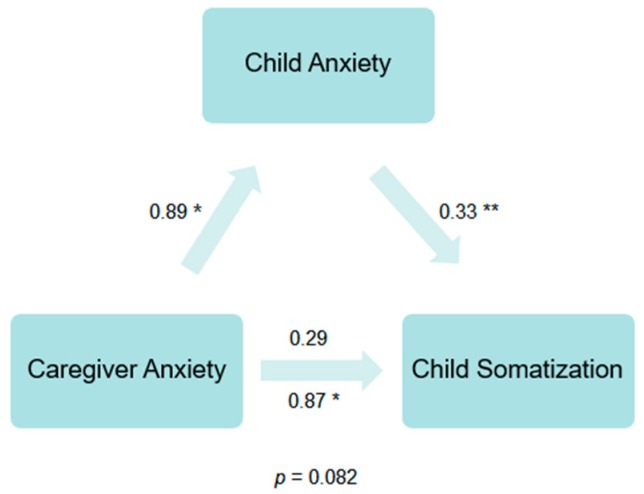
Cross-sectional indirect effect model for child anxiety predicting caregiver anxiety and child somatization. Notes: * beta is significant at the 0.05 level (2-tailed); ** beta is significant at the 0.01 level (2-tailed); The indirect effect was not significant (*p* = 0.082).

Caregiver anxiety significantly predicted greater degrees of child somatization (*b* = 0.87, *SE* = 0.41, *t* = 2.11, *p* = 0.035, 95% CI (0.06, 1.68)). The relation between caregiver anxiety and child anxiety was also significant (*b* = 0.89, *SE* = 0.44, *t* = 2.02, *p* = 0.044, 95% CI (0.03, 1.75)). Further, the relation between child anxiety and child somatization while controlling for caregiver anxiety was significant (*b* = 0.33, *SE* = 0.10, *t* = 3.41, *p* = 0.001, 95% CI (0.14, 0.51)). The indirect relation between caregiver anxiety and child somatization through child anxiety was not significant (indirect effect, *b* = 0.29, *SE* = 0.17, *t* = 1.74, *p* = 0.082, 90% CI (0.02, 0.56)). See Figure 1.

## 4. Discussion

This is the first study to our knowledge that examined the relationships between caregiver distress, child anxiety, and pain-related outcomes such as somatization, pain, and functional disability amongst youth with FAPD. Aside from examining relationships between caregiver distress, child anxiety, and child pain-related outcomes, the present study also examined the potential role of child anxiety as a mediator of the relationship between caregiver distress and child FAPD outcomes. That is, the indirect relation between caregiver anxiety and child somatization via child anxiety was examined. This work expands upon our prior study which found that child pain catastrophizing mediates the association between caregiver responses to pain (particularly caregiver encouragement of child symptoms) and functional disability in youth with FAPD conditions [19] in several ways. This was accomplished by: (1) exploring caregiver mental health versus caregiver responses to child symptoms, the prior of which is known to be associated with poor mental health and health-related outcomes of youth [11], (2) studying the role of child anxiety versus child pain catastrophizing as a potential mediator, which is important given that child anxiety is associated with higher pain and pain-related disability in youth with FAPD both in cross-sectional investigations [27,34], and over the long-term [28], and is associated with poorer response to pain-focused psychological interventions [30] and (3) specifically examining how both caregiver anxiety and child anxiety impacts child somatization, which is a detrimental outcome of FAPD [6,13,14]. 

This work also expands upon previous research on the associations between maternal distress, child mental health symptoms, and pain-related outcomes in caregivers with Irritable Bowel Syndrome (IBS) and their children. This prior work revealed that maternal distress (e.g., anxiety, depression, and somatization) and child distress in youth were related. However, this research did not examine specific pediatric FAPD diagnoses (the focus was on mothers diagnosed with IBS), utilized mother’s reports only, and did not examine additional FAPD outcomes such as functional disability [26]. The current study expands upon this work by examining relationships between distress of the primary caregiver, child anxiety, and child FAPD-related outcomes. Studying youth with FAPD diagnoses is clearly representative of youth receiving care at GI subspecialty clinics [28] and therefore the results are clinically meaningful. 

When examining relationships between caregiver distress, child anxiety, and child pain-related outcomes, it was found that caregiver anxiety, child anxiety, and child somatization were all significantly related to one another. This finding was generally consistent with our initial hypothesis that higher levels of general caregiver distress (e.g., stress, anxiety, and depression), child anxiety, and broad FAPD outcomes (e.g., somatization, functional disability, and pain) would be inter-related. However, other caregiver distress variables (e.g., depression and stress) were not related to FAPD outcomes and child anxiety was only associated with the FAPD outcomes of functional disability and somatization, not pain. 

Given the relationship between caregiver anxiety, child anxiety, and child somatization, we evaluated the potentially mediating role of child anxiety on the relationship between caregiver anxiety and child somatization. It was found that the indirect relation between caregiver anxiety and child somatization through child anxiety was not significant. Although child anxiety, caregiver anxiety, and child somatization are all inter-related, greater levels of child anxiety did not indirectly link higher caregiver anxiety to increased child somatization in youth with FAPD at a statistically significant level. While previous research has shown that child anxiety and pediatric FAPD are both associated with increased somatization, these factors had not yet been studied together in pediatric FAPD, which is a substantive contribution of this investigation. 

We also found that increased child anxiety was related to higher levels of child functional disability. Our finding is consistent with previous research demonstrating that anxiety symptoms in pediatric FAPD are associated with pain-related disability [18,28,29]. Indeed, youth with recurrent abdominal pain conditions such as FAPD show decreased participation in daily activities (e.g., school attendance decreases, less participation in hobbies, etc.), which may be reflected by increased functional disability levels [12,39]. 

Within our sample, when examining primary caregivers, it was found that caregiver anxiety, stress, and depression were all significantly correlated with one another. This may be due to the structure of the DASS reflecting a tripartite model of the commonly co-occurring constructs of negative affect (depression), absence of positive affect (stress), and hyperarousal (anxiety) [40]. Interestingly, caregiver distress (e.g., anxiety, stress, and depression) was not related to caregiver gender. Although it is known that adult females generally report higher levels of anxiety and depression compared to adult males [41] these results suggest that primary caregivers of youth with FAPD (regardless of their gender) may experience multiple domains of caregiver distress and that these domains may be inter-related. Larger-scale investigation of male caregivers is warranted to better understand how these psychological factors impact males. 

The results of the present study demonstrate that there is a positive and significant relationship between child anxiety and pain-related adverse outcomes in FAPD. Specifically, child anxiety was related to increased functional disability (supporting prior research) and child somatization, the latter of which is a novel contribution of our research. Our results also demonstrate that there is a positive and significant relationship between caregiver anxiety and child somatization. Thus, it may be particularly important to screen for both child and caregiver psychological factors to better understand which youth may have the greatest level of pain-related impairment (e.g., somatization, functional disability) associated with their FAPD.

These results may also have implications for the psychological treatment of FAPD. Given that child anxiety was related to increased child functional disability, child somatization, and caregiver anxiety, this suggests it is essential to monitor and address child anxiety when working with youth with FAPD. Cunningham et al. (2018) examined the impact of a psychological treatment for youth with FAPD that targeted both pain and anxiety. Preliminary results indicated that targeting pain and anxiety through the intervention resulted in reductions in functional disability [42] which suggests that targeting child anxiety in youth with FAPD can lead to reductions in adverse pain-related outcomes. Additionally, given that caregiver anxiety was related to both child anxiety and child somatization, it may also be important to address caregiver anxiety when treating youth with FAPD. All of these factors are potentially modifiable and responsive to psychological interventions, such as cognitive-behavioral therapy, an evidence-based intervention for anxiety and FAPD [42,43]. 

### Future Research and Limitations

Future research conducted using longitudinal designs could be beneficial in enhancing understanding of the relationships between child anxiety, caregiver anxiety, and child pain-related outcomes in FAPD. Future research could also benefit from examining the relationships between caregiver distress, pain-related outcomes, and child anxiety in other chronic pain populations to see if similar results are obtained. 

Strengths of the current study include the recruitment and analysis of a fairly heterogeneous sample in terms of the gender of youth with FAPD. With 42% of these youths identifying as male, the current sample is more representative of community samples than other clinical studies that have over-represented females (e.g., 80% or more female sample, etc.) [17,44]. Including a larger percentage of males in our sample likely increases the generalizability of our results. We also included primary caregivers of both genders, although our sample was over-represented by female caregivers.

Despite the significant strengths of this study, limitations are also present which should be considered when interpreting the results. The sample for the current study was from a single geographic area (Midwest region of the United States). Similarly, participant ages were limited to ages 9–14, limiting the generalizability of the results. Another limitation is that the present sample was also predominantly Caucasian. Future work is needed to generalize these results to more racially and ethnically heterogeneous samples. Given our strong interest in broader child anxiety that would be more characteristic of clinical anxiety disorders (versus pain-specific anxiety-provoking cognitions), we did not include a measure of pain catastrophizing in this current study. We note that our research examined whether child anxiety accounts for caregiver distress in relation to pain-related outcomes, which we conceptualized to include somatization (consistent with other literature). However, we note that yet other research has explored somatization as a mediator between child anxiety and pain severity [45,46], and between coping with pain and pain severity [45]. We did not explicitly examine coping with pain in our investigation. We focus on somatization (versus pain severity) as an outcome given that it has been found to be an important indicator of not just pain severity, but maintenance and impact of FAPD (for a review see [47]). 

Overall, this paper provides a unique understanding of caregiver and child psychological factors and their combined impact on outcomes associated with pediatric FAPD. 

## Figures and Tables

**Table 1:** Percentage of Race/Ethnicity in Sample.

	Race/Ethnicity Percentage
**Race**	
Caucasian	89%
African-American	4.4%
Biracial	3.3%
American Indian/Alaskan Native	2.2%
Other	1.1%
**Ethnicity**	
Latino/Hispanic	3.3%

**Table 2:** Correlations Between Caregiver and Child Measures.

	Range	*M*	*SD*	1	2	3	4	5	6	7	8
1. VAS	0–10	3.8	16.2	-	0.19	0.27 *	−0.01	−0.01	−0.07	0.19	0.12
2. CSI	0–140	32.7	16.2	0.19	-	0.04	0.09	0.22*	0.13	0.36 **	0.09
3. FDI	0–60	18.2	8.5	0.27 *	0.04	-	0.12	0.15	0.18	0.21 *	0.21 *
4. DASS Dep	0–14+	3.3	3.8	−0.01	0.09	0.12	-	0.70 **	0.61 **	0.13	0.26 *
5. DASS Anx	0–10+	3.4	4.1	−0.01	0.22 *	0.15	0.70 **	-	0.67 **	0.25 *	0.19
6. DASS Stress	0–17+	6.6	4.4	−0.07	0.13	0.18	0.61 **	0.67 **	-	0.17	0.13
7. SCARED	0–82	34.6	16.8	0.19	0.36 **	0.21 *	0.13	0.25 *	0.17	-	0.04
8. Age	9–14	11.6	1.74	0.12	0.09	0.21 *	0.26 *	0.19	0.13	0.04	-

Note: * correlation is significant at the 0.05 level (2-tailed); ** Correlation is significant at the 0.01 level (2-tailed); *M* = Mean; *SD* = Standard Deviation; VAS = Visual Analog Scale; CSI = Children’s Somatization Inventory; FDI = Functional Disability Inventory; DASS Dep/Anx/Stress = Depression Anxiety Stress Scales; SCARED = Screen for Anxiety and Related Disorders.
